# LINC01322 may serve as a potential diagnostic marker for advanced stage tumors in renal cell carcinoma patients eligible for total nephrectomy

**DOI:** 10.1016/j.bbrep.2024.101843

**Published:** 2024-10-13

**Authors:** Amirhosein Maharati, Negin Taghehchian, Fatemeh Taghavinia, Alireza Golshan, Azadeh Aarabi, Mohammad Reza Abbaszadegan, Meysam Moghbeli

**Affiliations:** a Student Research Committee, Faculty of Medicine, Mashhad University of Medical Sciences, Mashhad, Iran; b Medical Genetics Research Center, Mashhad University of Medical Sciences, Mashhad, Iran; c Department of Urology, Faculty of Medicine, Mashhad University of Medical Sciences, Mashhad, Iran; d Kidney Transplantation Complications Research Center, Mashhad University of Medical Sciences, Mashhad, Iran

**Keywords:** Renal cell cancer, LINC01322, Diagnosis, Tumor marker

## Abstract

**Background:**

Renal cell carcinoma (RCC) is a common urological cancer globally and shows a favorable prognosis in early stages of the tumor progression. Due to the poor prognosis for metastatic RCC patients, it is crucial to explore the molecular biology of RCC progression to establish efficient diagnostic and therapeutic markers for these patients. Long non-coding RNAs (lncRNAs) have critical roles in regulation of tumor cell proliferation, migration, and apoptosis during RCC progression. For the first time in the present study, we assessed the LINC01322 RNA expression levels in RCC patients to introduce that as a potential tumor marker among these patients.

**Methods:**

we visualized LINC01322 expression data using the online tool Gene Expression Profiling Interactive Analysis (GEPIA2) across different cancers and normal tissues. Fifty fresh samples of RCC tumor tissues and their adjacent normal margins were collected to analyze the RNA expression of LINC01322 and its association with the clinicopathological features of RCC patients. The SYBR green method was used in real-time PCR to measure the LINC01322 RNA expression levels in RCC patients.

**Results:**

Based on in-silico analysis, we hypothesized that LINC01322 could be involved in RCC progression by interacting with VHL, thereby influencing the tumor microenvironment. There were significant increased levels of LINC01322 RNA expressions in advanced stage compared with primary stage tumors that were located in left kidney (p = 0.048). Left kidney that were undergone the total nephrectomy had significant higher levels of LINC01322 RNA expressions compared with tumors in right kidney (p = 0.045). There was a direct correlation between the levels of LINC01322 RNA expression and RCC tumor size.

**Conclusions:**

considering the substantial increase in LINC01322 RNA expression in advanced stage RCC tumors that are candidates for total nephrectomy; it could be suggested as a potential diagnostic indicator for high-risk patients. In-silico analysis also revealed that LINC01322 could be involved in regulation of tumor microenvironment during RCC progression by interacting with VHL. However, further investigations are needed to validate the potential link between LINC01322 and VHL during RCC progression. Evaluating the serum LINC01322 RNA levels in RCC patients is also necessary to use that as a diagnostic marker in clinical settings.

## Highlights

• We assessed the LINC01322 expression levels in RCC patients to introduce that as a potential tumor marker.
• LINC01322 can be suggested as a potential diagnostic indicator for high-risk RCC patients.
• In-silico analysis also revealed that LINC01322 regulated tumor microenvironment during RCC progression by interacting with VHL.

## 1 Introduction

Renal cell carcinoma (RCC) arises from the epithelial cells of the proximal tubules within the renal tubules [1,2]. There are 434,840 new diagnoses and 155,953 RCC related deaths globally [3]. RCC is primarily classified into papillary RCC (pRCC), clear cell RCC (ccRCC), and chromophobe RCC (chRCC), with ccRCC being the most prevalent, representing approximately 75 % of all RCC cases [4,5]. Surgical resection remains as the main treatment for RCC patients [6]. Given the limited efficacy of chemo-radiation therapy in RCC, early detection and intervention are crucial for improving patient outcomes [7]. RCC typically presents without specific symptoms and is often detected incidentally, resulting in late-stage diagnosis when the tumor has developed resistance to chemo radiotherapy or metastasized [8]. While recent advancements in molecular targeted therapy have improved the prognosis for RCC patients, achieving long-term survival remains a significant challenge [9,10]. There is still a lack of efficient biomarkers for early detection and prognosis in RCC patients. Despite the high prevalence of RCC, the molecular mechanisms underlying tumor initiation, progression, and metastasis has remained poorly understood. Both genetic and epigenetic factors are involved in the pathogenesis of RCC [11]. Therefore, focusing on the molecular biology of RCC progression can be a promising approach to overcome the complexity of RCC diagnosis and treatment.

Long non-coding RNAs (lncRNAs) are a subclass of transcripts exceeding 200 nucleotides in length that are used as biomarkers due to their tissue-specific expression patterns [12]. They have key roles in tumor progression by acting as oncogenes or tumor suppressors in various cancers [[13], [14], [15], [16]]. The expression level and functional roles of lncRNAs and their association with clinicopathological features have also been reported in RCC patients [17,18]. Microarray and high-throughput sequencing technologies have identified various lncRNAs that may serve as crucial regulators of RCC progression [19]. LINC01322 is a long intergenic non-coding RNA (lincRNA) that is located on the long arm of chromosome 3. It has been identified as a prognostic biomarker in bladder and lung cancers [20,21]. Another study has revealed that LINC01322 increased lung tumor cell proliferation and migration via stimulation of the JAK/STAT signaling pathway [22]. Despite the reports about the role of LINC01322 in various cancers, there is still not any report in RCC patients. Von Hippel-Lindau (VHL) exhibits somatic and epigenetic mutations in approximately 80 % of clear cell RCC (ccRCC) patients [23]. The proximity of LINC01322 to the VHL gene on chromosome 3, suggests the LINC01322 as a probable regulator of RCC progression. Therefore, in the present study we assessed the levels of LINC01322 RNA expressions in RCC patients to suggest that as a biomarker among these patients.

## 2 Methods

### 2.1 In silico validation

To validate the RNA expression of LINC01322, we visualized its expression data using the online tool Gene Expression Profiling Interactive Analysis (GEPIA2) (http://gepia2.cancer-pku.cn/) across different cancers and normal tissues. Then we extracted a list of the top 100 genes most closely related to LINC01322 from GEPIA2. These genes were imported into the STRING database to construct a protein-protein interaction (PPI) network, which we further refined using Cytoscape software [24]. Finally, we performed GO and KEGG enrichment analysis on the gene sets using the ShinyGO (http://bioinformatics.sdstate.edu/go/) web tool.

### 2.2 Patients

The study involved fifty RCC patients who underwent nephrectomy at Mashhad University of Medical Sciences between 2022 and 2024. Tissue samples were collected from both the tumor and the adjacent normal areas, with the normal tissue specifically taken from a spot 5 cm away from the tumor mass. None of the patients with RCC had received chemotherapy or radiotherapy prior to the surgery. The tumors were graded and staged with the Fuhrman grading system and TNM classification, respectively. Following the surgery, samples of tissue were preserved in RNA-later solution and frozen at −80 °C for future RNA extraction. Participants provided their consent forms following approval from the ethics committee at Mashhad University of Medical Sciences. This study followed the guidelines outlined in the Declaration of Helsinki.

### 2.3 RNA extraction and qRT-PCR

The extraction of RNAs from fresh frozen RCC and normal tissues was performed using the Total RNA extraction Kit (Parstous, Iran). The extracted RNA was assessed for purity by gel agarose electrophoresis and spectrophotometry. Following that, the Easy cDNA reverse transcription kit (Parstous, Iran) was employed to carry out cDNA synthesis using an oligo dT primer. The RNA expression levels of LINC01322 were assessed using the SYBR green technique in duplicate reactions on a Light Cycler for real-time PCR analysis. All data was normalized using GAPDH. The following primers were used: LINC01322 - forward 5′-GATGGCAGAGAAGACAAAGAGAAC-3′, reverse 5′-AACAACTGGATAGGGTAACAAAGC-3′; GAPDH - forward 5′-GGAAGGTGAAGGTCGGAGTCA-3′, reverse 5′-GTCATTGATGGCAACAATATCCACT-3′. The RNA expression levels of LINC01322 were assessed using the -ΔΔCT method. An increase in fluorescence intensity twice the baseline indicates up regulation, whereas a decrease below double the baseline indicates down regulation [25,26].

### 2.4 Statistical analysis

For statistical analyses, SPSS version 27.0.1 (SPSS, Chicago, IL) was used. The findings were presented as mean values along with a standard deviation (SD). The data distribution's normality was assessed with the Kolmogorov-Smirnov test. LINC01322 RNA expression levels were assessed for their correlation with various clinicopathological factors using chi-square/Fisher exact tests, ANOVA, and independent sample *t*-test, with a significance level set at P ≤ 0.05.

## 3 Results

### 3.1 Raw data preprocessing and gene selection

**Fig. 1:**
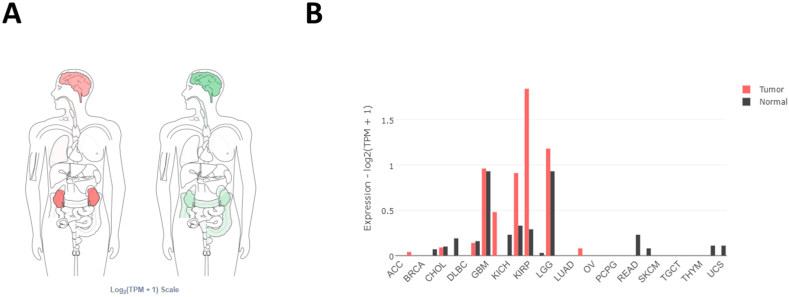
The validation of LINC01322 on the TCGA and Genotype-Tissue Expression (GTEx) samples. A) Bodymap: The bodymap shows the median expression of LINC01322 in different body tissues (Green = normal, red = tumor). B) Bar plot: The gene expression profile across all tumor samples and adjacent normal tissues. (For interpretation of the references to colour in this figure legend, the reader is referred to the Web version of this article.)

**Fig. 2:**
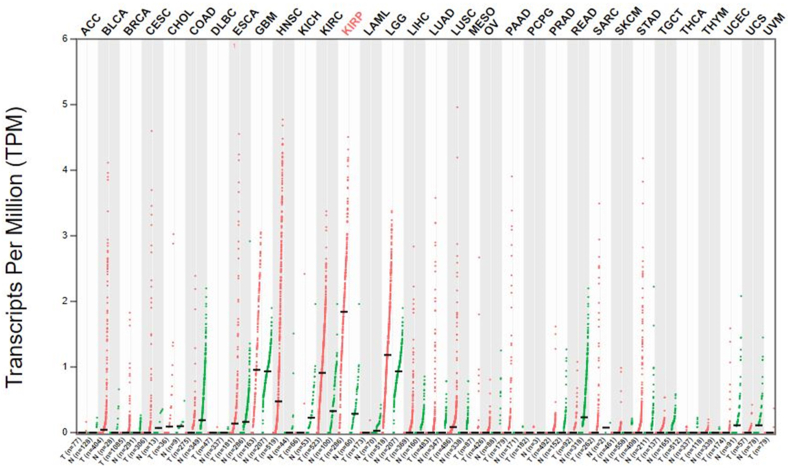
The expression profile of LINC01322 among all TCGA projects.

**Fig. 3:**
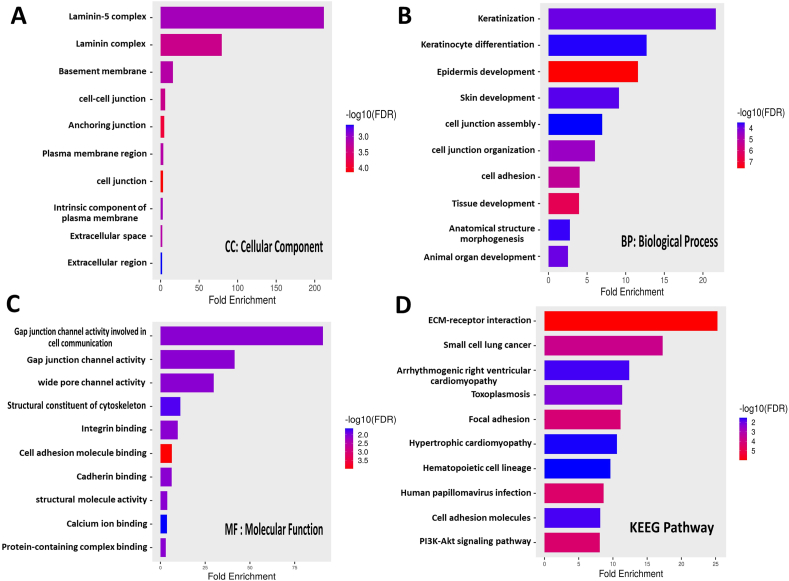
Enrichment analysis on the top 100 genes that are most related to LINC01322. A) Cellular component: represents that the genes were dominantly enriched in the laminin-5 complex. B) Biological process: reveals that genes are enriched in the keratinization and keratinocyte differentiation. C) Molecular function: these genes were mostly involved in the gap junction channel activity. D) Kyoto Encyclopedia of Genes and Genomes (KEGG) Pathway analysis: the LINC01322 related genes was involved in ECM-receptor interaction.

**Fig. 4:**
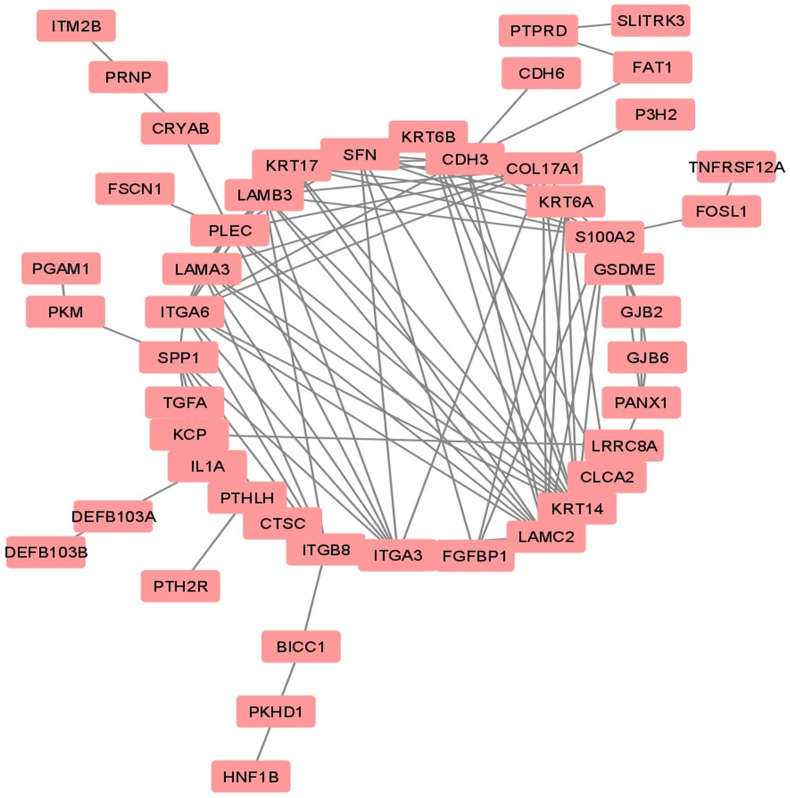
String Protein-Protein Interaction (PPI) network that indicates the interactions between LINC01322 related genes constructed by cytoscape.

We searched for LINC01322 on the GEPIA2 website and observed up regulation in brain and kidney tissues (Fig. 1A). There was a significant up regulation of LINC01322 in RCC tissues compared to adjacent normal tissues (Fig. 1B). Interestingly, LINC01322 was also up regulated in The Cancer Genome Atlas Program (TCGA) cohorts for pRCC, ccRCC, head and neck squamous cell carcinoma (HNSC), and glioblastoma multiforme (GBM) (Fig. 2). Additionally, we extracted the 100 genes most closely related to LINC01322 from GEPIA2 and conducted enrichment analysis using ShinyGO. This analysis revealed that these genes were significantly involved in various molecular functions, cellular components, biological processes, and KEGG pathways, including laminin-5 complex, keratinization and keratinocyte differentiation, gap junction channel activity, and ECM-receptor interaction (Fig. 3). Moreover, we constructed a PPI network with these LINC01322 related genes (Fig. 4). Therefore, these in-silico analyses suggest that LINC01322 may play a crucial role in RCC and its microenvironment.

### 3.2 Study population

The RCC population in our study included 24 (48 %) males and 26 (52 %) females. The patients ranged in age from 24 to 80 years, with a mean age of 55.88 ± 12.24 years. Women were younger than men (53.27 ± 2.46 vs. 58.71 ± 2.35 years). Tumors varied in size from two to 16 cm, with an average size of 5.45 ± 3.13 cm. Most of the tumors were located in the left kidney (27/50, 54 %), had a low grade (39/50, 78 %), and were detected in early stages (44/50, 88 %). Complete nephrectomy was the primary surgical treatment for 66 % of RCC patients (33 out of 50). Table 1 offers a summary of the clinicopathological and demographic characteristics of RCC patients.

**Table 1:** Correlation between the levels of LINC01322 expressions and clinicopathological features of RCC patients.

	Total	LINC01322 under expression	Normal LINC01322 expression	LINC01322 over expression	*p-value*
**Patients**	50	10 (20 %)	31 (62 %)	9 (18 %)	
**Mean age (years, mean ± SD)**	55.88 ± 12.24	55.20 ± 5.32	56.16 ± 2.09	55.67 ± 3.19	0.591
**Size (cm, mean ± SD)**	5.45 ± 3.13	3.84 ± 0.46	5.77 ± 0.58	6.14 ± 1.26	0.695
**Sex**					0.446
Male	24 (48 %)	4 (40 %)	17 (54.8 %)	3 (33.3 %)	
Female	26 (52 %)	6 (60 %)	14 (45.2 %)	6 (66.7 %)	
**Location**					**0.045**
Left	27 (54 %)	5 (50 %)	14 (45.2 %)	8 (88.9 %)	
Right	23 (46 %)	5 (50 %)	17 (54.8 %)	1 (11.1 %)	
**Grade**					0.919
Low grade (I/II)	39 (78 %)	8 (80 %)	24 (77.4 %)	7 (77.8 %)	
High grade (III/IV)	11 (22 %)	2 (20 %)	7 (22.6 %)	2 (22.2 %)	
**Lymph node metastasis**					0.067
Yes	1 (2 %)	–	–	1 (11.1 %)	
No	49 (98 %)	10 (100 %)	31 (100 %)	8 (88.9 %)	
**Stage**					**0.048**
I/II	44 (88 %)	10 (100 %)	28 (90.3 %)	6 (66.7 %)	
III/IV	6 (12 %)	–	3 (9.7 %)	3 (33.3 %)	
**Nephrectomy**					0.419
Total	33 (66 %)	5 (50 %)	21 (67.7 %)	7 (77.8 %)	
Partial	17 (34 %)	5 (50 %)	10 (32.3 %)	2 (22.2 %)	

### 3.3 Clinicopathological features and LINC01322 expression

**Fig. 5:**
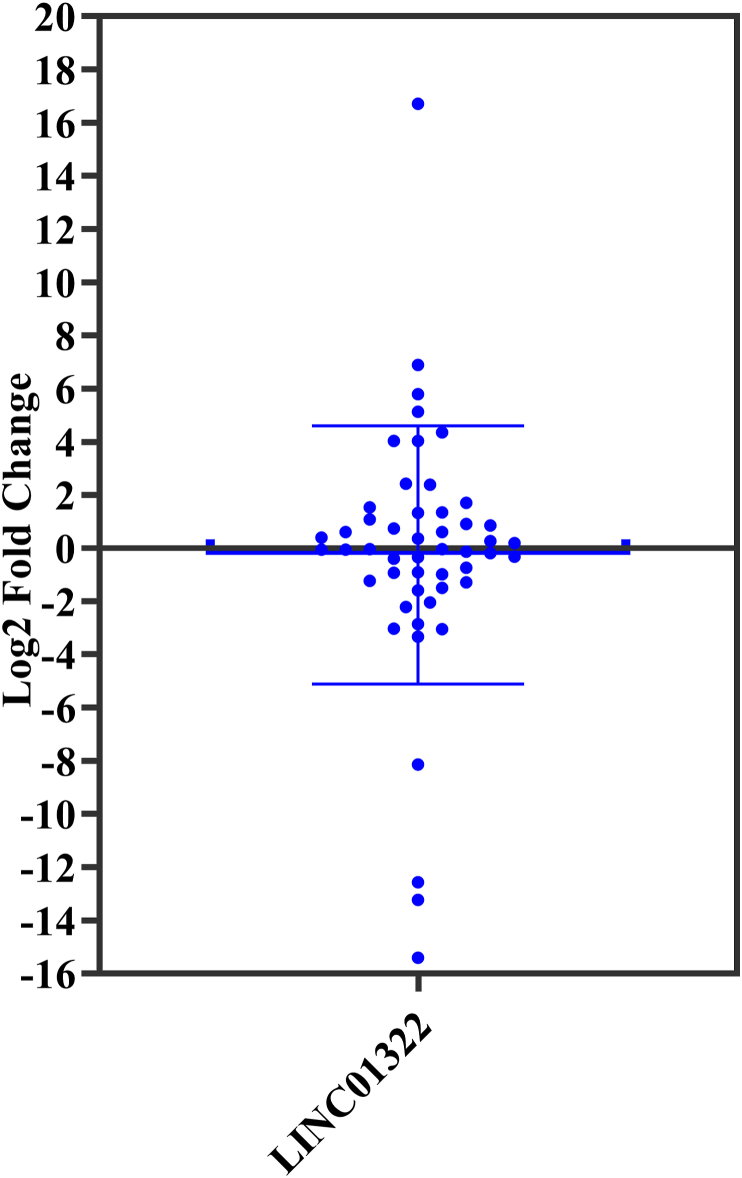
Descriptive analysis of relative LINC01322 expression in RCC patients.

**Fig. 6:**
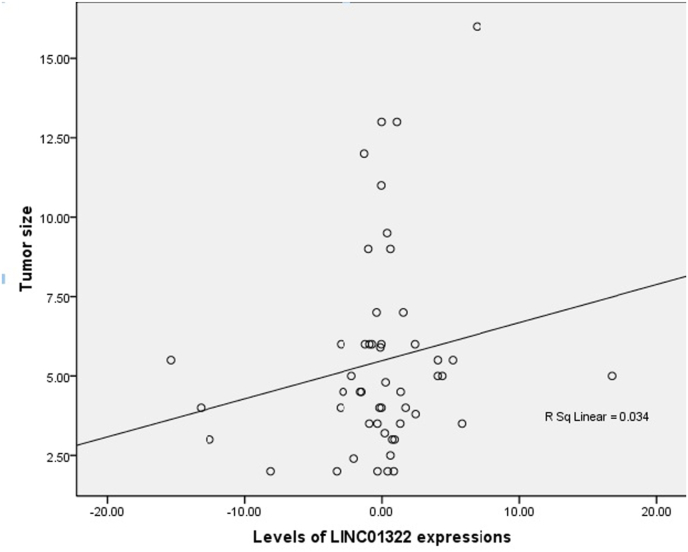
Linear regression analysis of LINC01322 expression relative to tumor size in RCC patients.

Levels of LINC01322 RNA expression were evaluated in fifty RCC patients. LINC01322 showed down regulation in 10 (20 %) and up regulation in 9 (18 %) of the fifty RCC patients. The fold changes of LINC01322 varied from −15.41 to 16.71, averaging −0.26 ± 4.58 (Fig. 5). Male RCC patients exhibited reduced LINC01322 RNA expression levels in comparison to females (−1.19 ± 1.08 vs. 0.61 ± 0.86, fold changes). High-grade (III/IV) RCC tumors exhibited reduced levels of LINC01322 RNA expressions in comparison to low-grade (I/II) tumors (−1.49 ± 1.88 vs. 0.09 ± 0.71, fold changes). There were significant increased levels of LINC01322 RNA expressions in advanced stage compared with primary stage tumors that were located in left kidney (2.74 ± 1.27 vs. −0.04 ± 1.33, fold changes) (p = 0.048). Left kidney that were undergone the total nephrectomy had significant higher levels of LINC01322 RNA expressions compared with tumors in right kidney (1.55 ± 1.60 vs. −1.11 ± 0.82, fold changes) (p = 0.045). There was a direct correlation between the levels of LINC01322 RNA expressions and tumor size (Fig. 6). Interestingly, the tumors with LINC01322 up regulation had bigger size compared with tumor with LINC01322 down regulation (6.14 ± 1.26 vs. 3.84 ± 0.46, cm).

## 4 Discussion

Renal cancer as a prevalent malignancy within the urinary tract system among Iranian population has imposed a significant burden on the public health system [27]. The primary treatment option is surgical resection of the local tumor or nephrectomy. However, late-stage diagnosis of RCC or post-surgery recurrence often is treated with chemotherapy and radiotherapy, which have limited efficacy and led to reduced overall survival in these patients. Furthermore, immunotherapy has not yet yielded promising results [28]. Therefore, early detection of RCC, along with a deeper understanding of its molecular pathology and the mechanisms underlying cancer progression and metastasis, is crucial for identifying therapeutic targets and improving patient prognosis. LncRNAs are involved in tumor cell growth, energy metabolism, chemo resistance, and metastasis [[29], [30], [31]]. They can act as oncogenes or tumor suppressors by recruiting chromatin remodeling complexes to gene promoter regions [32]. Cytoplasmic lncRNAs regulate mRNA stability or translation, thereby influencing downstream signaling pathways [33,34]. Deregulation of different lncRNAs such as MRCCAT1, HEIRCC, GIHCG, SARCC, and lncRNA-ATB has been also reported in RCC tissues which were correlated with prognosis [[35], [36], [37], [38]]. LINC01322 is classified as a RUS (“RNA upstream of Slitrk3”) gene that influences nearby genes involved in neurogenesis. Inhibition of LINC01322 can arrest neuronal differentiation and proliferation while promotes apoptosis [39,40]. VHL syndrome is a hereditary syndrome characterized by the development of hemangioblastoma and clear cell RCC [41]. VHL gene plays a crucial role in this syndrome; which functions as a tumor suppressor by regulating hypoxia-inducible factors [42]. Loss of VHL protein function results in increased cell proliferation, angiogenesis, and tumor progression [[43], [44], [45]]. The most frequent genetic alteration identified in RCC is the biallelic deletion or loss of heterozygosity on chromosome 3p that affects the VHL gene [46]. Given the proximity of LINC011322 and VHL on chromosome 3, as well as the role of LINC011322 in neurogenesis, it is possible that LINC011322 plays a significant role in ccRCC and brain tumors, which are associated with VHL syndrome, through its interaction with VHL. There was LINC01322 up regulation in lung cancer that was associated with poor prognosis [22].

We assessed the RNA expression level of LINC01322 using large databases such as TCGA and found that this gene is up regulated in RCC. Then we assessed the levels of LINC01322 RNA expressions in a sub-population of Iranian RCC patients. We observed that there were reduced levels of LINC01322 RNA expressions in high-grade RCC tumors in comparison with low-grade tumors. There were significant increased levels of LINC01322 RNA expressions in advanced stage compared with primary stage tumors that were located in left kidney. Left kidney that were undergone the total nephrectomy had significant higher levels of LINC01322 RNA expressions compared with tumors in right kidney. RCC tumors with LINC01322 up regulation had bigger size compared with tumor with LINC01322 down regulation. Additionally, enrichment analysis suggested that LINC011322 may play a pivotal role in RCC and its tumor microenvironment, as the genes related to LINC011322 was involved in extracellular matrix (ECM) interactions. VHL/HIF/VEGF pathway is recognized as a key driver in RCC tumor development [47]. HIF up regulates the expression of hypoxia-inducible factors such as stromal cell-derived factor 1 (SDF1), CXC chemokine receptor 4 (CXCR4), VEGF, cyclin D1, PDGF-β, c-Met, and TGF-α [48]. These factors are primarily secreted by cancer-associated fibroblasts (CAFs), which are crucial cells in the tumor microenvironment. Additionally, these genes are implicated in ECM functions, including cell surface interactions, collagen synthesis, and adhesion [49,50]. Therefore, genes that regulate the expression of VHL and its downstream targets are involved in modulating tumor-induced hypoxia and ECM remodeling of tumor cells. Based on in-silico analysis, we also hypothesize that LINC01322 could be involved in RCC progression by interacting with VHL, thereby influencing the tumor microenvironment.

## 5 Conclusions

Since, there was significant LINC01322 up regulation in advanced stage RCC tumors that were undergone the total nephrectomy, it can be proposed as a diagnostic marker in high risk cases. There was also a direct correlation between the levels of LINC01322 RNA expressions and RCC tumor size. In-silico analysis also showed that LINC01322 can be involved in RCC progression by interacting with VHL that regulates tumor microenvironment. However, functional studies are required to confirm the probable association between LINC01322 and VHL during RCC progression. It is also required to assess the RNA levels of LINC01322 in serum samples of RCC patients to suggest that as a reliable diagnostic marker in clinics.

## CRediT authorship contribution statement

**Amirhosein Maharati:** Writing – original draft, Methodology. **Negin Taghehchian:** Methodology. **Fatemeh Taghavinia:** Writing – original draft, Methodology. **Alireza Golshan:** Methodology. **Azadeh Aarabi:** Methodology. **Mohammad Reza Abbaszadegan:** Visualization, Validation. **Meysam Moghbeli:** Writing – review & editing, Visualization, Validation, Supervision, Project administration, Funding acquisition, Conceptualization.

## Ethics approval and consent to participate

All the experiments were performed according the declaration of Helsinki. All the patients have filled the informed consent forms that have been confirmed by the ethics committee of Mashhad University of medical sciences (#4030881).

## Funding

This research did not receive any specific grant from funding agencies in the public, commercial, or not-for-profit sectors.

## Declaration of competing interest

The authors declare that they have no known competing financial interests or personal relationships that could have appeared to influence the work reported in this paper.
